# West Ham and Eastern General Hospital Dinner

**Published:** 1913-11-29

**Authors:** 


### West Ham and Eastern General Hospital Dinner.

A RECORD IN THREE RESPECTS.

On Thursday, last week, the annual dinner of the West Ham and Eastern General Hospital was held at the Hamilton Hall, Great Eastern Hotel, Mr. Lionel A. Martin, a director of Messrs. Henry Tate and Sons, Ltd., of Silvertown and Liverpool, presiding.

This function was of extreme interest to all interested in the institution owing to the fact that it was a record in more ways than one—there was a record attendance of over 300 people, there was a record subscription list totalling upwards of £6,000, and there was a record collection for the Christmas Tree Fund for the children, which was taken in cash during the dinner. Mr. Martin stated in his speech that the hospital was in a very poor district, that the medical staff was second to none, and their work most excellent, and that the hospital was efficiently and economically managed and deserved help of all charitably inclined, and pointed out the urgent need of further extension, reading a most convincing letter from Sir William Tate, who had given a cheque for £1,600 towards the proposed Nurses' Home, and mentioning that Mr. Edwin Tate had given a cheque for £900 to complete the £2,500 estimated cost of land and buildings. Mr. Martin also mentioned that £700 had been promised by various donors towards equipment.

Mr. T. A. Cook, chairman of the hospital, in acknowledging the toast of "The Hospital," after congratulating those present on having the advantages of the help of a chairman so widely known in commercial affairs of international importance, and having acknowledged the very generous cheques of Sir William Tate, Bart., and Mr. Edwin Tate, proceeded to point out how all concerned in the hospital were constantly eager to secure the fullest efficiency possible; that there could be no hesitation that the needs of the district were not only larger than the institution was able to cope with, but were growing annually. He made an earnest appeal for funds to prevent the necessity of legacies and other capital moneys being spent in maintenance, or, as he expressed it, "on bread and butter."

The Mayor of West Ham, Alderman William Crow, referred to the hospital as one of the leading institutions of the borough, and one of which every burgess should be proud. The musical programme was under the direction of Mr. Geo. Briance. It is interesting to note that over £40,000 has been spent during the last few years in the extension of the above hospital, which, with its 110 beds, supplies the more urgent needs of a district containing approximately as many inhabitants as some of our large provincial cities.

